# Health shocks in Sub-Saharan Africa: are the poor and uninsured households more vulnerable?

**DOI:** 10.1186/s13561-018-0210-x

**Published:** 2018-10-13

**Authors:** Esso-Hanam Atake

**Affiliations:** Department of Economics, University of Lome, Lome, Togo

**Keywords:** Vulnerability to poverty, Health shocks, Health insurance, Poverty, Fertility, Sanitation, Education, Sub-Saharan Africa

## Abstract

**Background:**

In developing countries, health shock is one of the most common idiosyncratic income shock and the main reason why households fall into poverty. Empirical research has shown that in these countries, households are unable to access formal insurance markets in order to insure their consumption against health shocks. Thus, in this study, are the poor and uninsured households more vulnerable from health shocks? We investigate the factors that lead to welfare loss from health shocks, and how to break the vulnerability from health shocks in three Sub-Saharan Africa (SSA) countries, namely, Burkina Faso, Niger and Togo.

**Methods:**

This study focusses on 1597 households in Burkina Faso, 1342 households in Niger and 930 households in Togo. A three-step Feasible Generalized Least Squares (FGLS) method was used to estimate vulnerability to poverty and to model the effects of health shocks on vulnerability to poverty.

**Results:**

The estimates of vulnerability show that about 39.04%, 33.69%, and 69.03% of households are vulnerable to poverty, in Burkina Faso, Niger, and Togo respectively. Both interaction variables, ‘health shocks and wealth’ and ‘health shocks and access to health insurance’ had a significant negative effect on reducing household’s vulnerability to poverty. Poverty is the leading cause of economic loss from health shocks as the poorer cannot afford the purchase of sufficient quantities of quality food, preventive and curative health care, and education. We found that lack of health insurance coverage had a significant effect by increasing the incidence of welfare loss from health shocks. Moreover, household size, type of health care used, gender, education and age of the head of the household as well as the characteristics of housing affect vulnerability to poverty.

**Conclusion:**

Our findings suggest that for the poor households, reduction of user fees of health care at the point of service or expansion of health insurance could mitigate vulnerability to poverty. Other challenges—birth control policy, adequate sanitation facilities and a universal basic education program—need to be addressed in order to reduce significantly the effects of health shocks on vulnerability to poverty in SSA.

## Background

Households facing health shocks may find themselves permanently impoverished due to loss of income associated with illness and the cost of access to health care [1]. Health shocks are a sudden deterioration of an individual’s health, caused by illness and/or injury [2]. These health shocks, defined as unpredictable diseases that deteriorate the health status, are some of the most important factors associated with poverty [3]. “Health shocks and their associated costs have both short and long term impacts on households welfare” [2, 4]. In the short term, households facing health shocks are forced to substitute consumer and production spending for health care [4]. In the long term, net flows of investment in productive activities tend to decrease [4]. In the process, there is a possibility that health shocks may lead households to poverty or make them even poorer in the near future.

Vulnerability to poverty refers to the risk that the household will fall into poverty due to a standard of living that is below the poverty line. According to Novignon et al. [2], vulnerability to poverty refers to the probability that a household or an individual, whether currently poor or not, will become poor in the near future. The poorest households, such as those in Sub-Saharan French-Speaking Africa countries (SSAF) may be the most vulnerable to health shocks. The lowest-ranked countries in the Human Development Index (HDI) of 2016 are SSAF countries, namely: Togo (166th), Benin (167th), Cote d’Ivoire (171st), Mali (175th), DRC (176th), Guinea (183rd), Burkina Faso (185th), Chad (186th), Niger (187th) and the Central African Republic (188th) out of 188 countries. In these poorest countries, it is possible to envisage the poor becoming poorer and the non-poor becoming poor due to health shocks. This study attempts to explore this prospect by analyzing whether the poor and uninsured households are more vulnerable to poverty due to health shocks in three Sub-Saharan Africa (SSA) countries: Burkina Faso, Niger and Togo.

Francophone West Africa countries, Burkina Faso, Niger and Togo are part of the CFA Franc (West African Economic and Monetary Union) and share common cultural traditions. The poverty line varies from one country to another. The latest data show that it is lower in Burkina and higher in Togo (Table 1). It is 153,530 CFA francs per year and per inhabitant (i.e., $US 274.16 per year) in Burkina compared to 344,408 CFA francs (or $US 615.01 per year per inhabitant) in Togo. About half of the population lives below the poverty line and the direct household payments as a percentage of private health expenditure exceed 80% (Table 1).

**Table 1:** Poverty and health funding profile

Country	National poverty line (CFA Franc)	% of State budget allocated to health	Direct payments by households in percentage of private health expenditure (%)	Private health expenditure in % of total health expenditure	Population health insurance cover (%)
Burkina-Faso	153,530	12.50	82.69	38.40	Less than 2
Niger	182,635	6.58	83.85	40.00	Less than 3
Togo	344,408	3.62	84.6	48.6	7.66

With regard to health financing, the share of the general budget allocated to health sector in these countries is low. As a matter of fact, it is below the 15% of the annual general budget recommended at the Abuja summit in 2000. Consequently, the financing of the social sectors, particularly the health sector, is still very low. It is remarkably low in Togo, where in 2015, the percentage of the State budget allocated to health was about 3.62%. Apart from Togo, less than 3% of the population has health insurance cover: less than 2% in Burkina Faso, less than 3% in Niger and 7.66% in Togo. Since 2012, Togo implemented the National Health Insurance Scheme (NHIS). NHIS is a mandatory health insurance which covers civil servants, civil servant retirees, and their dependents [5]. Private and informal sectors workers do not have access to NHIS. Despite the importance of informal sector workers in the total labor force (90.4%), they are excluded from this health insurance scheme. As a result, it covers only 4% of the Togolese population [6]. Mutual insurance companies and private insurance companies, respectively cover 1.66% and 2.00% of the Togolese total population [6]. Only in January 2018, the government of Burkina Faso adopted a schedule of operationalization of the universal health insurance plan (RAMU) which paves the way for the imminent adoption of the decrees creating the management bodies (RAMU). In Niger, there is a bill of law to make universal coverage a developmental priority. At the moment, despite the will of the authorities, universal health coverage is not yet effective.

In the SSA countries where insurance markets are inadequate and imperfect, household consumption may be subject to shocks [7, 8]. In fact, in the absence of formal insurance mechanisms, health shocks could have negative economic consequences on households. For example, Wagstaff [9] provide evidence that in Vietnam health shocks are associated with a reduction in consumption, for uninsured households [9]. The degree of vulnerability depend largely on whether the household has formal health insurance, or is covered by a fee-waiver program [9]. Since SSA countries have moved towards universal health coverage, understanding the impact of health shocks is crucial in explaining to policymakers the challenges of designing social protection programmes for health. Furthermore, literature has shown that the major problem is general poverty in those countries and not only health shocks as a leading cause of vulnerability [10, 11]. Poverty may be the main cause of ill health and economic loss, in SSA.

The present study tries to answer the following research questions: Are the poor and uninsured households are more vulnerable to poverty due to health shocks? What are the factors that lead to welfare loss from health shocks? How to break the vulnerability related to health shocks in SSA countries? We hypothesize that health shocks, access to health insurance and wealth are not mutually exclusive. Our goal is to help decision-makers increase capability levels and reduce risk in order to combat vulnerability, thereby preventing households from gliding into poverty as a result of health shocks. This study could also contribute to the promotion of universal access of health services through social protection.

## Methods

### Data and variables

#### Data sources

The data was collected from national statistics offices in each country. In the case of Niger, we used data from the National Survey on Vulnerability to Food Insecurity of Households carried out in 2014. For Burkina Faso, the data was sourced from the Continuous Multi-Sector Survey conducted in 2014. With respect to Togo, the data originated from the survey of the Core Welfare Indicators Questionnaire conducted in 2015. This study focusses on households that had reported illness in the 4 week preceding the surveys and covers 1597 households in Burkina Faso, 1342 households in Niger and 930 households in Togo. These surveys used similar questionnaires, methodology, and sampling frame. They collected similar information on all categories of expenditure (e.g. food and non-food expenditure).

#### The main variables

##### Dependent variable

Household total food and non-food expenditures are used as a dependent variable to estimate the expected consumption [2]. For each food item, households were asked about the amount they had used for purchases, home consumption of their own production, and other sources in the reference period. The reference period for foods items differ depending on the type of food: some foods (e.g. beef, chicken) are consumed occasionally (once or twice a month), while others (e.g. rice, lentils) are consumed much more frequently. Non-food consumption is measured annually since some of the items are purchased occasionally. The measure of non-food consumption expenditure includes items such as soap, housing repairs, clothing, kerosene, batteries, etc. but excludes expenditure on irregular items (e.g. dowry, marriage, costs of legal and court cases, etc.). We aggregated all expenditures in these two broad categories and valued it using the price quoted by the household (unit value). The price variation is at the item-household-year level. Given that households buy foodstuffs of different quality (e.g. coarse rice, fine rice, etc.) and that it is difficult to monitor the price of each quality, we used the data reported by the households. However, where there were some inconsistencies (for example, a very high or very low value), we used the village level median price to convert the stated quantity into a monetary value. To ensure that the expenditures in different regions were comparable, we used regional deflators calculated from price collections of various commodities in the regions. Lastly, once the various items of expenditure were annualized, the presence of outliers was detected and these values were re-estimated using the median value (item by item, area by area). A minute proportion of expenditures (less than 0.5%) was re-estimated.

#### Independent variables

##### Variables of interest

The literature points to the importance of the choice of the health shock variable in analyzing the impact of the disease on vulnerability to poverty. In some case, it was found that the results can vary significantly depending on how the health shock is measured. “A household is said to face a health shock when an illness or injury weakens the health status of its member and generates a welfare loss for the household” [12]. Health shocks affect not only the patient but the entire household in many ways. It may be illness/injury, hospitalization, death, presence of chronic illness in the household, utilization of health care, ability limitation to perform work-related activities connected to health status, disability of the head of household, etc. [13]. Unfortunately, in developing countries, households are generally unable to access formal insurance markets to insure their consumption against such shocks [14]. These households are vulnerable since they are not able to smooth consumption. The uninsured health shocks - adverse events that cost individuals and households dearly – could be proxy by hospitalization of a working age household member [9, 15], death of an employed household member [9], number of days of regular activity lost due to illness or injury [13], occurrence of illness and/or injury [2], recent sizeable drop in the body mass index of the household head [9], etc. In this study, we used as a proxy of health shocks the household’s health status [2]. This variable was approximated by standardizing the occurrence of a disease and/or injury (i.e., the number of individuals in the household who have had a disease and/or injury during the 4 weeks preceding the survey divided by the total number of individuals in the household) [2]. This choice is justified not only by the availability of data but also by the fact that in the absence of formal insurance mechanisms, the incidence of health shock in a household would lead to significantly increased out-of-pocket payments and is also likely to increase the incidence of poverty.

##### Variables of control

The hygienic conditions of the household were introduced into the model to capture the household’s health status as well. This variable is approximated by the presence or absence of safe drinking water and hygienic latrines within the household [2]. We assumed that households without access to safe drinking water and hygienic latrines do not have good hygienic conditions [2, 16]. Furthermore, we used the Strauss and Thomas [17] conceptual framework to define other control variables that are key determinants of the level of household well-being, namely, characteristics of the household (Location, access to health insurance, household size, and wealth) [13] and characteristics of the household head (age, gender, and education status) [13].

Household size is measured by the number of individuals that make up the household. The education level of the head of household was a four-modality dummy variable (uneducated, primary, secondary, higher education). Insurance variable was a dummy with a value of 0 if household has access to health insurance and 1 otherwise. Location dummy variable was 1 if rural and 0 urban residence. Gender was a dummy variable that takes value 0 if the head of the household is a man and 1 otherwise. Likewise, the age of the head of household was a four-modality dummy variable. The household characteristics are approximated by access to electricity (dummy variable) and owner of the housing (dummy variable). Finally, households were categorized into 5 wealth quintiles (from the poorest households to the richest ones).

In the first model, interaction variables (which is the product of the variable of interest and the interaction variable) are included in the regression. In fact, we introduced interaction effects between health shock variable and wealth and insurance variables, as we hypothesized that they are not mutually exclusive. The interaction effects between health shock, wealth, and access to health insurance were included as other explanatory variables. In the second model, we introduced the interaction variables individually. These two different models help also to infer structural validity from coefficients. Indeed, we investigate through our models how certain “core” regression coefficient estimates behave when the regression specification is modified [18]. We test robustness by dropping and adding covariates, in order to determine whether we have estimated effects of interest, or only predictive coefficients [18].

As far as the missing variables are concerned, they relate exclusively to control variables. Regardless of the country and the control variables under consideration, the percentage of data loss do not exceed 3.1%. We analysed the descriptive statistics of other household characteristics with missing data and compared the mean of the variables of households with missing data and those without missing data. As the averages were not very different, it was concluded that the missing data was not significant enough to cast doubts on the results obtained.

### Vulnerability to poverty threshold

Referring to the literature [2, 19, 20], we use the poverty vulnerability threshold of 0.5. Two main reasons were used in the literature to justify the choice of 0.5. Novignon et al. [2] assume that it is more intuitive to say that a household with a 50% probability of falling into poverty in the next period is vulnerable to poverty. Households with an estimated value of vulnerability to poverty of 0.5 or more are considered vulnerable to poverty. Secondly, Pritchett et al. [20] justify the choice of 0.5 by supporting the idea that when a household with current consumption levels equal to the poverty line faces a zero-medium shock, there is 0.5 vulnerability. In the limit, if the time horizon is close to zero, “in current poverty” and being “currently vulnerable” coincide [20]. It can fall below the poverty line at any time.

### Statistical methods

In the literature, three principal approaches are used to access vulnerability: vulnerability as expected poverty (VEP), vulnerability as low expected utility (VEU), and vulnerability as uninsured exposure to risk (VER) [19, 21]. These approaches have similar characteristics since they construct a model that predicts a measure of welfare [19]. The VEP is defined as the probability that the expected consumption expenditure of a household will fall into poverty in the future [22]. VEU is defined by referring to the difference between the utility derived from a certain level of consumption that would be its equivalent and to which or beyond which the household would not be considered vulnerable [23]. VER assesses welfare loss in the absence of effective risk management tools [19, 23].

We used in this study Chaudhuri, Jalan, and Suryahadi [22] and Christiaensen and Subbarao [24] VEP approaches defined as the probability that a household will fall into poverty in the future in such a way that a household’s vulnerability corresponds to the probability that the level of consumption of a household in the future will fall below the consumption poverty line. Two main reasons justify this choice [2]. First, these estimation methods focus on panel data. This is the period approach and the component approach. However, in the absence of panel data, in SSAF countries, VEP, unlike VEU allows the use of cross-sectional data in estimating vulnerability to poverty [2, 22, 25, 26]. Despite the limitations of purely cross-sectional data, an analysis of vulnerability to poverty via this data is potentially informative with such an approach. Secondly, the VEP approach allows an ex-ante evaluation of future poverty compared to the VER variant which allows an ex-post evaluation of the scope of the negative shock causing a loss of well-being.

#### Estimation of vulnerability to poverty

Chaudhuri et al. [22] and Christiaensen and Subbarao [24] define welfare in terms of consumption so that the vulnerability of household *h* at time *t* - *V*_*ht*_ - is the probability that the household’s level of consumption at time *t* + 1 (*C*_*ht* + 1_) will be below consumption poverty line, *z*. In other words:

1 $$ {V}_{ht}=\Pr \left(\ln {C}_{h,t+j}\prec \ln z\right) $$

They assume that consumption is determined by the following stochastic process:

2 $$ \ln {C}_{ht}=\beta {X}_h+{\varepsilon}_h $$

Where, ln*C*_*ht*_is the logarithm of per capita consumption expenditure of household *h*, *X*_*h*_ a vector of the characteristics of the household (e.g. location, characteristics of the head of household, shocks, etc.), *β*the vector of the parameters to be estimated, and *ε*_*h*_ a zero-mean random term.

The use of cross-sectional data requires the formulation of certain assumptions [2]. First, the*ε*_*h*_ random term is log-normally distributed, implying that *C*_*h*_ consumption expenditure is also log-normally distributed. Second, the structure of the economy is stable over time, excluding the possibility of aggregate shocks (i.e. unanticipated structural changes in the economy). The first hypothesis makes it possible to estimate the probability that a household with *X*_*h*_ characteristics will be poor (household vulnerability level). The latter hypothesis implies that uncertainties about future consumption arise solely from uncertainty about idiosyncratic shocks that the household will experience in the future. Vulnerability estimates should therefore be interpreted with the assumption that current economic structures will prevail, at least in the near future.

The vulnerability to poverty of household *h* with the *X*_*h*_ characteristics can be calculated using the estimated coefficients of eq. 2, such that:

3 $$ {\overset{\hat{\mkern6mu} }{V}}_h=\Pr \left(\ln {C}_{h,t+1}\prec \ln z\left|{X}_h\right.\right)=\phi \left(\ln z-{X}_h\overset{\hat{\mkern6mu} }{\beta}\overset{\hat{\mkern6mu} }{\sigma}\right) $$

Where ${\overset{\hat{\mkern6mu} }{V}}_h$ is the vulnerability to estimated poverty, *ϕ*(.) is the cumulative density of the standard normal distribution, and $\overset{\hat{\mkern6mu} }{\sigma }$ is the standard error from eq. 2.

A simple functional form is used to establish the relationship between the variance of the consumption function and household characteristics:

4 $$ {\sigma}_{\varepsilon, h}^2={X}_h\theta $$

#### Presence of heteroscedasticity

In the survey data, some respondents may provide more specific answers than others. “As there is probably some error in the measurement of consumption, this may have resulted in significant overestimation of the variance of consumption, and thus of vulnerability” [27]. An advantage of the estimation using the Three-Step Feasible Generalized Least Squares (FGLS) method developed by Amemiya [28] to estimate *β* and *θ* in this paper is that it provides a consistent estimate of the true variance of consumption even when consumption is measured with measurement errors [27]. Amemiya [28] proved its consistency and asymptotic normality. In the FGLS estimate, the unknown matrix is replaced by a consistent estimator. FGLS also provides a robust estimate through the verification of autocorrelation in the *ε*_*h*_‘s.

The estimation steps are described as follows:

First, eq. (2) is estimated using the ordinary least squares method. The *ε*_*h*_ estimated residues of eq. (2) are thus used to estimate the following equation by OLS:

5 $$ {{\overset{\hat{\mkern6mu} }{\sigma}}^2}_{ols,h}={X}_h\overset{\hat{\mkern6mu} }{\theta }+{\overset{\hat{\mkern6mu} }{\eta}}_h $$

The ${X}_h\overset{\hat{\mkern6mu} }{\theta }$ predicted values of this auxiliary regression are used to transform eq. (5).

6 $$ \frac{{{\overset{\hat{\mkern6mu} }{\sigma}}^2}_{ols,h}}{X_h\overset{\hat{\mkern6mu} }{\theta }}=\frac{X_h}{X_h\overset{\hat{\mkern6mu} }{\theta }}\theta +\frac{\eta_h}{X_h\overset{\hat{\mkern6mu} }{\theta }} $$

The estimation of eq. (6) by the OLS results in an asymptotically efficient FGLS estimator($\overset{\hat{\mkern6mu} }{\theta }$_*FGLS*_). It can be demonstrated that ${X}_h\overset{\hat{\mkern6mu} }{\theta }$_*FGLS*_ is an efficient estimator of ${\sigma}_{\varepsilon, h}^2$_,_which is the variance of the idiosyncratic component of household consumption. Equation (2) is also transformed with the standard error of $\overset{\hat{\mkern6mu} }{\theta }$_*FGLS*_ as follows:

7 $$ {\overset{\hat{\mkern6mu} }{\sigma}}_{\varepsilon, h}=\sqrt{X_h{\overset{\hat{\mkern6mu} }{\theta}}_{FGLS}} $$

8 $$ \frac{\ln {C}_h}{{\overset{\hat{\mkern6mu} }{\sigma}}_{\varepsilon, h}}=\left(\frac{X_h}{{\overset{\hat{\mkern6mu} }{\sigma}}_{\varepsilon, h}}\right)\beta +\frac{\varepsilon_h}{{\overset{\hat{\mkern6mu} }{\sigma}}_{\varepsilon, h}} $$

The OLS estimate of eq. (8) gives a consistent and asymptotically efficient estimate of β. The *β*_*FGLS*_ and *θ*_*FGLS*_ estimates allow a direct estimation of the log of expected consumption (presented in eq. 9) and the expected variance of the log of consumption (presented in eq. 10).

9 $$ E\left[\left(\ln {\overset{\hat{\mkern6mu} }{C}}_h\left|{X}_h\right.\right)\right]={X}_h\overset{\hat{\mkern6mu} }{\beta } $$

10 $$ Var\left[\left(\ln {\overset{\hat{\mkern6mu} }{C}}_h\left|{X}_h\right.\right)\right]={\overset{\hat{\mkern6mu} }{\sigma}}_h^2={X}_h\overset{\hat{\mkern6mu} }{\theta } $$

Assuming that consumption is logically distributed, vulnerability to poverty is estimated as follows:

11 $$ {\overset{\hat{\mkern6mu} }{V}}_h=\phi \left(\frac{\ln z-{X}_h{\overset{\hat{\mkern6mu} }{\beta}}_{FGLS}}{\sqrt{X_h{\overset{\hat{\mkern6mu} }{\theta}}_{FGLS}}}\right) $$

#### Time horizon

The literature show that the time horizon and welfare are quite arbitrary [19]. Hoddinott and Quisumbing [19] emphasized that household could be poor next year, in 10 years’ time, or in old age. We think that the time horizon should be any period in the future [2]. It is not certain and there is a high probability that a household or individual will become poor exactly one period and/or year after health shocks. The time horizon was therefore specified in this study as *t* + *j* instead of *t* + 1, with *j* ≥ 1 [2, 22, 24].

## Results

### Descriptive statistics

Table 2 shows that about 43% of heads of households are between 15 and 49 years of age. While in Burkina Faso and Niger, about 90% of household heads have a maximum level of primary education; in Togo about 42% have at least a secondary education level. These contradictory statistics can also be observed with respect to household size. In Burkina Faso and Niger, more than 40% of households have an average household size of at least 7 people. In Burkina Faso, for example, about 23.36% of households have an average household size of more than 10 people. In Togo, on the other hand, more than 75% of households have an average size of less than 6 persons. In addition, the average proportion of sick individuals per household is estimated at 47.08%, 23.40% and 60.57% respectively in Burkina Faso, Niger and Togo. Lastly, it’s important to emphasize that almost all households do not have access to health insurance (99.87%, 99.98, and 93.76 in Burkina Faso, Niger, and Togo respectively).

**Table 2:** Summary of descriptive statistics

Demographic and sanitary characteristics	Burkina-Faso (%)	Niger (%)	Togo (%)
Proportion of sick individual per household	47.08	23.40	60.57
Proportion of household access to health insurance	0.13		6.24
Location			
Urban	36.63	34.13	63.76
Rural	63.37	65.87	36.24
Head Education			
None	78.34	79.36	30.32
Primary	10.90	10.36	26.77
Secondary	7.39	8.20	42.15
High school	2.38	2.09	0.75
Head Age			
15–29	9.33	7.08	14.42
30–49	34.38	42.85	46.29
50–64	30.00	33.38	23.90
65 -	26.30	16.69	15.39
Head gender			
Male	76.46	74.89	66.99
Female	23.54	25.11	33.01
Household size			
1–3	18.19	12.40	35.70
4–6	34.80	45.06	40.22
7–9	23.64	27.47	16.24
10 –	23.36	15.08	7.35
Access to safe drinking water			
Yes	73.07	52.76	62.80
No	26.93	47.24	37.20
Access to sanitation facilities			
Yes	40.26	25.67	60.22
No	59.74	74.33	39.78
Number of household	1597	1342	930

### Vulnerability to poverty

The average estimated vulnerability to poverty was at 39.04% in Burkina Faso, 33.69% in Niger and 69.03% in Togo (Table 3). It is relatively high in Togo (coastal country) compared to Burkina Faso and Niger (Sahelian countries). While in Burkina Faso and Niger, the vulnerability to population ratio as a result of health shocks was about 39.42% and 33.31% respectively, in Togo it was estimated at 69.03%. Generally speaking and regardless of the country vulnerability to poverty is observed very high for the uninsured and the poor households. Especially, in Togo, all the poor households were vulnerable to poverty due to health shocks (Table 4). Moreover, vulnerability to poverty was very high among households in rural areas (49.17%, 45.94% and 84.89% respectively in Burkina Faso, Niger and Togo), compared to those in urban areas (21.11%, 9.45% and 59.60% in Burkina Faso, Niger and Togo respectively). It is interesting to focus on the result showing that vulnBurkina Faso and Niger, male-headed households were more vulnerable to poverty than female-headed households. Among health problems, illness or accidents encountered by households over the past 4 weeks, the main sources of vulnerability to poverty were malaria, skin disease, accidents and injuries and diarrhea and stomach aches. With regard to the type of recourse, households which patronize small size public hospitals (peripheral health units, Maternal Child Health Centre, etc.) are highly vulnerability to poverty (30.19%, 36.92% and 71.83% respectively in Burkina Faso, Niger and Togo).

**Table 3:** Vulnerability to poverty profile by country

	Burkina Faso		Niger		Togo	
	Mean Vulnerability	Vulnerability to population ratio	Mean Vulnerability	Vulnerability to population ratio	Mean Vulnerability	Vulnerability to population rati
Total	39.04	39.42	33.69	33.31	68.74	69.03
Health insurance						
Insurance coverage	0.00	0.00	5.71	9.09	40.61	36.21
Not insurance coverage	39.06	39.45	33.95	33.53	70.61	71.22
Gender of household head						
Male	40.45	41.20	37.00	37.30	67.00	67.42
Female	39.92	33.02	22.71	20.00	72.29	72.31
Location						
Urban	21.11	21.56	9.45	7.50	59.60	60.88
Rural	49.17	49.51	45.94	46.34	84.89	83.38
Household size						
1–3	8.18	7.58	6.57	0.00	48.27	47.89
4–6	33.62	33.27	26.49	26.44	75.78	76.74
7–9	46.20	47.23	44.89	46.04	87.45	88.08
10 –	63.90	65.49	57.20	58.10	86.78	86.30
Illness and treatment						
Malaria	31.44	31.25	36.61	36.57	63.35	66.38
Diarrhea/Intestinal worms	18.61	17.78	37.00	31.03	48.50	50.00
Dental disease	44.30	50.00	23.35	20.00	48.64	48.64
Skin disease	28.43	33.33	38.13	38.46	79.56	79.56
Eyes disease	4.71	0.00	26.88	22.22	77.22	77.22
Accident and injuries	0.00	0.00	37.18	32.43	79.00	79.00
Type of health care sought						
Religious/ NGO	19.91	20.00	29.48	33.33	42.85	45.00
Small size public Hospital	30.19	30.26	36.92	36.81	71.83	72.05
Large size public hospitals	14.46	12.50	16.16	16.67	65.91	66.02
University Teaching Hospitals	0.00	0.00	11.41	11.11	63.43	66.67
Private hospitals	10.01	9.52	15.97	13.64	64.48	64.44
Number of household	1551		1342		930

**Table 4:** The vulnerable and the poor in Togo (%)

	Vulnerable (%)	Non-vulnerable (%)	Total
Very Poor	100	0.00	100
Poor	100	0.00	100
Mean	100	0.00	100
Rich	96.76	3.24	100
Very Rich	0.35	99.65	100
Number of household	642	288	930

Pearson Chi2 (4): 893.65

Probability: 0.000

Lastly, the Chi-2 independence test (Table 4) shows that there is a relationship between poverty and vulnerability to poverty (*P* value = 0.000).

### Determinants of vulnerability to poverty

We found that health shocks, household wealth and access to health insurance were not mutually exclusive. Both interactions variables, ‘health shocks and wealth’ and ‘health shocks and insurance’ had a significant negative effect on reducing household’s vulnerability to poverty. Thus, the poorer and uninsured households were highly vulnerable to poverty due to health shocks. These results remained robust to introduction of interaction effects in the model individually as well as simultaneously (Table 6). The results in Table 5 show that there is a negative relationship between the reduction of vulnerability to poverty and household size, regardless of the country under consideration. Vulnerability to poverty is higher when the size of the household is high. Furthermore, the results show that the level of education is one of the main determinants of vulnerability to poverty. The results in Table 5 show that heads of households with a high level of education are less likely to be vulnerable to poverty. It is also important to note that the characteristics of household housing are key determinants of vulnerability to poverty. Similarly, households with access to good hygiene and safe drinking water are less likely to be vulnerable to poverty. Finally, vulnerability to poverty was found to be lower for households with less number of ill members (Table 6).

**Table 5:** Model 1: factors leading to vulnerability to poverty from health shocks

Variable	Burkina Faso		Niger		Togo	
	Ex-ante mean consumption	Ex-ante variance consumption	Ex-ante mean consumption	Ex-ante variance consumption	Ex-ante mean consumption	Ex-ante variance consumption
Health shocks*Insurance	−1.19*** (−1.30—1.08)	− 005 (− 0.08–0.07)	− 0.582*** (− 0.79--0.37)	0.06 (− 0.13–0.25)	− 1.13*** (− 1.26--1.00)	− 0.26*** (− 0.35--0.17)
Health shocks*Wealth	0.33*** (0.31–0.36)	0.007 (− 001–0.02)	1.31*** (1.24–138)	0.31*** (0.24–0.37)	0.42*** (0.39–0.45)	0.04*** (0.03–0.06)
Gender						
Female	− 0.16*** (− 0.14—0.04)	− 0.02 (− 0.06–0.01)	0.01 (− 0.03–0.06)	0.03 (− 0.01–0.08)	− 0.15*** (− 0.22—0.08)	0.003 (− 0.05–0.05)
Head Education						
Primary	0.08** (0.005–0.15)	− 0.03 (− 0.07–0.02)	0.10*** (0.04–0.17)	− 0.002 (− 0.06–0.06)	0.09** (0.005–0.18)	− 0.11** (− 0.17–0.04)
Secondary	0.22*** (0.13–0.31)	0.01 (− 0.05–0.07)	0.20*** (0.13–0.28)	0.002 (− 0.07–0.07)	0.09** (0.002–0.19)	− 0.07** (− 0.13--0.004)
High school	0.54*** (0.39–0.7)	0.001*** (− 0.10–0.10)	0.48*** (0.34–0.62)	0.31*** (0.18–0.44)	− 0.08 (− 0.44–0.27)	− 0.06 (− 0.31–0.19)
Head Age						
30–49	− 0.06 (− 0.14–0.02)	−0.03 (− 0.09–0.02)	−0.08*** (− 0.17—0.00)	0.001 (− 0.07–0.07)	0.10** (− 0.001–0.20)	0.01 (− 0.06–0.08)
50–64	−0.11*** (− 0.20--0.03)	−0.04 (− 0.1–0.01)	0.001 (− 0.08–0.08)	−0.01 (− 0.09–0.06)	0.20*** (0.09–0.31)	−0.03 (− 0.11–0.04)
65 -	−0.13*** (− 0.22--0.05)	−0.01 (− 0.07–0.05)	0.04 (− 0.05–0.13)	−0.004 (− 0.08–0.08)	0.12* (− 0.005–0.24)	−0.04 (− 0.13–0.04)
Household size						
4–6	−0.43*** (− 0.51--0.15)	−0.06** (− 0.11--0.01)	−0.03 (− 0.15–0.08)	0.05 (− 0.06–0.16)	−0.15*** (− 0.24--0.07)	0.003 (− 0.06–0.06)
7–9	−0.59*** (− 0.68--0.50)	−0.03 (− 0.08–0.03)	−0.10 (− 0.23–0.04)	0.11* (− 0.02–0.23)	−0.37*** (− 0.48--0.25)	0.01 (− 0.07–0.09)
10 –	−0.75*** (− 0.84--0.66)	−0.06* (− 0.11–0.003)	−0.17 (− 0.32—0.01)	0.17** (0.03–0.30)	−0.42*** (− 0.55--0.28)	0.02 (− 0.08–0.11)
Household Characteristics						
Location						
Rural	−0.19*** (− 0.24—0.14)	−0.03 (− 0.06–0.01)	−0.14*** (− 0.2—0.07)	0.04 (− 0.02–0.09)	−0.22*** (− 0.30—0.14)	0.03 (− 0.02–0.09)
Good Hygiene	0.11*** (0.06–0.17)	0.01 (− 0.02–0.04)	0.17*** (0.11–0.23)	−0.01 (− 0.03–0.04)	0.19*** (0.12–0.27)	−0.02 (− 0.07–0.03)
Access to safe water	0.16*** (0.11–0.21)	−0.004 (− 0.035–0.03)	0.004 (− 0.04–0.05)	−0.01 (− 0.06–0.03)	0.07* (0.001–0.14)	0.03 (− 0.01–0.08)
Number of household	1551	1551	1292	1292	929	929

*, **, *** Significant at 10%, Significant at 5%, Significant at 1%

(): 95% Confidence Interval

**Table 6:** Model 2: factors leading to vulnerability to poverty from health shocks

Variable	Burkina Faso		Niger		Togo	
	Ex-ante mean consumption	Ex-ante variance consumption	Ex-ante mean consumption	Ex-ante variance consumption	Ex-ante mean consumption	Ex-ante variance consumption
Health Insurance						
Not insurance coverage	0.06 (−0.42–0.55)	0.12 (− 0.13–0.38)	−0.32^c^ (− 0.47--0.16)	0.03 (− 0.05–0.10)	0.09^b^ (0.005–0.18)	0.01 (− 0.04–0.06)
Gender						
Female	−0.10^c^ (− 0.14—0.07)	−0.01 (− 0.03–0.004)	0.03 (− 0.05–0.06)	0.002 (0.77—0.01)	−0.10^c^ (− 0.15—0.05)	0.01 (− 0.02–0.03)
Proportion of sick individual	−0.09^a^ (− 0.10--0.02)	−0.15^a^ (− 0.03–0.00)	−0.07^a^ (− 0.14–0.02)	0.01 (− 0.003–0.02)	−0.05 (− 0.13 --0.25)9	0.03 (− 0.02–0.07)
Head Education						
Primary	− 0.018 (− 0.05–0.03)	−0.01 (− 0.03–0.01)	0.04 (− 0.01–0.08)	0.004 (0.76−− 0.02)	0.02 (−0.04–0.08)	-0.02 (− 0.05–0.01)
Secondary	−0.00 (− 0.06–0.05)	−0.01 (− 0.04–0.02)	0.11^c^ (0.06–0.17)	0.05^c^ (0.02–0.08)	0.07^b^ (0.01–0.14)	−0.01 (− 0.05–0.02)
High school	0.50^c^ (0.40–0.59)	0.03 (− 0.02–0.08)	0.35^c^ (0.24–0.46)	0.02 (0.57−− 0.04)	0.09 (− 0.14--0.33)	-0.04 (− 0.18–0.10)
Head Age						
30–49	−0.07^c^ (− 0.12--0.02)	−0.00 (− 0.03–0.02)	−0.12^c^ (− 0.18—0.05)	0.015 (− 0.01–0.05)	0.04 (− 0.02–0.11)	−0.01 (− 0.05–0.03)
50–64	− 0.10^c^ (− 0.15--0.04)	−0.01 (− 0.04–0.02)	−0.10^c^ (− 0.16--0.04)	0.03^b^ (0.001–0.06)	0.10^c^ (0.03–0.17)	−0.02 (− 0.06–0.02)
65 -	−0.13^c^ (− 0.18--0.08)	−0.003 (− 0.03–0.02)	−0.09^c^ (− 0.16−− 0.03)	0.02 (0.01–0.05)	0.03 (− 0.05–0.11)	-0.03 (− 0.08–0.01)
Household size						
4–6	−0.20^c^ (− 0.25--0.15)	−0.06^c^ (− 0.09--0.04)	−0.44^c^ (− 0.52--0.35)	0.05^b^ (0.04–0.02)	−0.13^c^ (− 0.18--0.07)	−0.0004 (− 0.03–0.03)
7–9	−0.19^c^ (− 0.25--0.13)	−0.05^c^ (− 0.08--0.02)	−0.77^c^ (− 0.87--0.67)	0.05^a^ (0.05--0.00)	−0.21^c^ (− 0.29--0.14)	−0.001 (− 0.04–0.04)
10 –	−0.20^c^ (− 0.26--0.14)	−0.06^c^ (− 0.10--0.03)	−0.12^c^ (− 1.23—1.01)	0.066^b^ (0.01–0.12)	−0.28^c^ (− 0.37--0.19)	−0.01 (− 0.06–0.04)
Household Characteristics						
Location						
Rural	−0.10^c^ (− 0.14—0.07)	−0.02^a^ (− 0.03–0.00)	−0.08^c^ (− 0.12—0.03)	−0.04 (− 0.03–0.02)	−0.16^c^ (− 0.21—0.11)	0.01 (− 0.02–0.04)
Good Hygiene	0.04^c^ (0.01–0.07)	0.002 (− 0.01–0.02)	0.06^b^ (0.01–0.1)	0.03 (− 0.02–0.02)	0.08^c^ (0.03–0.13)	0.01 (− 0.02–0.04)
Access to safe water	0.04^c^ (0.01–0.07)	0.003 (− 0.01–0.02)	− 0.01 (− 0.05–0.02)	− 0.01 (0.24–0.03)	0.03^a^ (− 0.01–0.08)	0.02^a^ (− 0.002–0.05)
Wealth quintiles						
Second poorest	0.36^c^ (0.32–0.41)	− 0.02^a^ (− 0.04–0.002)	0.56^c^ (0.51–0.61)	− 0.03^b^ (− 0.06—0.01)	0.58^c^ (0.50–0.65)	− 0.11^c^ (− 0.15—0.06)
Middle	0.66^c^ (0.62–0.70)	−0.02^a^ (− 0.04–0.002)	0.88^c^ (0.83–0.93)	−0.04^c^ (− 0.05—0.01)	0.94^c^ (0.87–1.02)	−0.109^c^ (− 0.15—0.06)
Second wealthiest	0.98^c^ (0.94–1.02)	− 0.01 (− 0.04–0.01)	1.17^c^ (1.12–1.23)	−0.03^b^ (− 0.06—0.01)	1.29^c^ (1.22–1.37)	−0.11^c^ (− 0.15—0.07)
Wealthiest	1.67^c^ (1.62–1.72)	0.08^c^ (0.05–0.10)	1.70^c^ (1.64–1.76)	0.02 (− 0.01–0.05)	1.94^c^ (1.86–2.01)	−0.05^b^ (− 0.09—0.002)
Number of household	1551	1551	1292	1292	929	929

^a^, ^b^, ^c^ Significant at 10%, Significant at 5%, Significant at 1%

(): 95% Confidence Interval

## Discussion

The results in Table 3 shown that about 68.74%, 33.69%, and 39.04% of Togolese, Nigerians and Burkinabe respectively are vulnerable to poverty. The highly vulnerability to poverty from health shocks in Togo could be related to lives of poor households especially in rural areas. About 73.9% of Togo’s rural population lives below the poverty line making it one of the world’s poorest countries [29]. Poverty and vulnerability are widespread. The 2013 Demographic and Health Survey showed that in Togo more than 28% of children aged 6–59 months were suffering from chronic malnutrition and 6.5% from acute malnutrition. Other studies showed that the vulnerability of Togolese children is exacerbated by their lack of access to health and education [29]. In this context, occurrence of illness and/or injury could increase significantly vulnerability to poverty. These results corroborate the findings of International Monetary Fund on poverty and vulnerability in Togo which showed that if no action is taken to improve living conditions in Togo, the poverty incidence could reach 81.8% [29]. Our findings highlight the fact that it is not appropriate to focus exclusively on the current incidence of poverty in the implementation of various poverty reduction programs and projects. It is therefore important to take into account the current and future dimensions of poverty in all anti-poverty policies [2, 30]. With this in mind, since the results of our study shown that health shocks drive high proportion of households into poverty, policymakers in SSA countries should make explicit analyses of vulnerability to poverty dimensions in their poverty reduction strategies.

The most interesting result of our study is that poverty and access to health insurance were the keys determinants of household’s vulnerability to poverty due to health shocks. The poorer households faced further augmentation in economic loss due to health shocks. Poverty is the leading cause of economic loss due to health shocks as the poorer cannot not afford to purchase sufficient quantities of quality food, preventive and curative health care and education. Comparison of the findings with those of other studies confirms that poverty is the main cause of ill health and economic loss, in Sub-Saharan Africa [10, 11]. But, welfare loss is also related to other factors related to poverty, such as access to health insurance. We found that lack of health insurance coverage had a significant effect in increasing the incidence of welfare loss from health shocks. In absence of health insurance coverage, households compromise their consumption to meet worse health status expenditures. This study particularly highlighted this problem in Togo where a highly proportion of poorer and uninsured households was vulnerable. Atake and Amendah [5] found that in Togo, at the 40% threshold, health care cost represents 60.95% of insured households’ total monthly non-food expenditure: suggesting gaps in the coverage. Health coverage policies for the poorer households and indigents are largely insufficient and porous [5]. User fees not only denied the poorer households access to quality healthcare, but also lead to welfare loss. Our findings suggest that specific focus needs to be given to the poor and uninsured households to protect them against economic loss due to health shocks. An implication of this is the possibility that for the poor households among whom ill health and economic loss from health shocks tends to be concentrated, reduction of user fees at the point of service or expansion of health insurance could mitigate welfare loss.

Others important findings were that household size, type of health care used, gender, education and age of the head of the household as well as the characteristics of housing affect vulnerability to poverty.

Our results showed that household size is an important factor in the analysis of vulnerability to poverty in SSA countries. These results confirm those in SSA countries such as Ghana and Nigeria, where vulnerability to poverty increases with increasing household size [2, 31]. In SSA countries, we hypothesize that household size contributes significantly to vulnerability to poverty through health shocks and several channels of transmission. In countries where more than half of the households consist of more than 7 people, investments in health such as care during childbirth, pre-and post-natal care, child growth monitoring, maternal health, care for the elderly, etc. are obviously expensive and lead to vulnerability to poverty, in a context where there is no universal health coverage. The large size of households invariably reduces the well-being of the members of the household, which, de facto contributes to increased vulnerability to poverty [32–34]. It is clear from our results that the fertility policy which is currently being discussed/developed within the Economic Community of West African States (ECOWAS) should highlight the impacts of health shocks associated with high birth rates on vulnerability to poverty.

Furthermore, contrary to our expectations, it were small public hospitals (dispensaries, peripheral care units, maternal child health centre etc.) that contribute most to vulnerability to poverty compared to large public hospitals and private hospitals. It should be noted that low or no income is one of the main constraints to health care access in these countries [35]. Small public hospitals are frequented by poor households, generally from the rural areas. These households’ level of poverty is such that any additional health care expenditure is synonymous with impoverishment. As a result, self-medication behaviors, delays in consulting health center, and recourse to public health centers with affordable first aid costs have been observed [35, 36]. As the smaller public health center is the closest health facility to the population, it has the highest attendance rate for consultations regardless of the standard of living. When health care is needed but delayed or not achieved, individuals find themselves in poor health. This leads to income losses and high health care costs, both of which contribute to vulnerability to poverty [37]. The level of income that is an important factor in health care utilization significantly affects vulnerability to poverty [38]. These results confirm that universal coverage, at least for first aid and particularly for the poorest sections of the population, is one of the main factors directly related to vulnerability to poverty.

The results also showed that households headed by illiterate persons were the most affected by vulnerability to poverty. The higher the education level of the head of the household, the less vulnerable the household was to poverty. These findings are similar to those of Adepoju and Okunmadewa [31] who showed that in Nigeria, vulnerable heads of households are those who are generally uneducated or have at most primary education. These results can be attributed to the fact that people with a high level of education are more likely to have decent jobs and therefore a source of income that can mitigate the impacts of health shocks on vulnerability to poverty. Education thus appears as a solid defense against vulnerability to poverty [12].

In addition, our results are contradictory as far as the effect of gender on vulnerability to poverty is concerned. While in Burkina Faso and Togo, the likelihood of vulnerability to poverty increased among female-headed households, it was not significant in Niger. This contradiction has also been reported in the literature [39, 40].

Lastly, the study of household characteristics and vulnerability to poverty consists of identifying the link between access to safe drinking water and sanitation facilities. The results showed that households without access to safe drinking water and sanitation facilities were the most vulnerable to poverty. Inadequate provision of safe drinking water and sanitation, in terms of both quantity and quality contributes significantly to increased vulnerability to poverty [41]. These basic social services are directly linked to the households’ conditions of hygiene and their quality of life. The lack thereof negatively impacts the health status of households and leads to high vulnerability to poverty. It is important to encourage and promote public policies on access to safe drinking water and decent sanitation facilities for all segments of the population.

The main limitation of this study is the lack of long-term panel data that would have provided intertemporal consumption expenditure for the assessment of vulnerability to poverty. As pointed out by Novignon [2], this panel data would have provided information on the same households over time. One of the major shortcomings of this study is the inability to control the possible existence of the problems of endogeneity due to lack of adequate instruments in the available databases. In this study, this problem may exist in the sense that while health status can affect vulnerability to poverty, an opposite relationship may also exist [2]. Despite these limitations, this study identifies key messages regarding the impact of health policies on vulnerability to poverty in SSA countries. Moreover, vulnerability and poverty reinforce each other. Indeed, poverty is a source of vulnerability (poor people are more likely to fall badly sick or to be affected by political events) and repeated exposure to downturns reinforces poverty.

## Conclusion

The objectives of this study were to assess the effects of health shocks on vulnerability to poverty and identify the main factors contributing to this vulnerability in Sub-Saharan countries, namely, Burkina Faso, Niger and Togo. Our findings showed that both interactions variables, ‘health shocks and wealth’ and ‘health shocks and insurance’ had a significant negative effect on reducing household’s vulnerability to poverty. Poverty and lack of health insurance coverage were the leading causes of economic loss due to health shocks. Our findings suggest that for the poor households, reduction of user fees of health care at the point of service or expansion of health insurance could mitigate vulnerability to poverty. The evidence from this study suggests that health policies that address vulnerability to poverty in this region must be supported by a sound population/fertility program. Furthermore, the effects of health shocks on vulnerability to poverty can be mitigated by first focusing on education, which must be seen in this region as a solid defense against poverty. Secondly, programs for access to safe drinking water and sanitation must be strengthened because good hygiene conditions lead to reduced vulnerability to poverty.

## Abbreviations

ECOWAS: Economic Community of West African States
FGLS: Generalized Least Squares
HDI: Human Development Index
SSA: Sub-Saharan African
VEP: Vulnerability as Expected Poverty
VER: Vulnerability as Uninsured Exposure to Risk
VEU: Vulnerability as Expected Utility
WAEMU: West African Economic and Monetary Union
